# Bi-allelic variants in MTMR5*/SBF1* cause Charcot-Marie-Tooth type 4B3 featuring mitochondrial dysfunction

**DOI:** 10.1186/s12920-021-01001-1

**Published:** 2021-06-12

**Authors:** Beatrice Berti, Giovanna Longo, Francesco Mari, Stefano Doccini, Ilaria Piccolo, Maria Alice Donati, Francesca Moro, Renzo Guerrini, Filippo M. Santorelli, Vittoria Petruzzella

**Affiliations:** 1 Child Neurology Unit, Meyer Children’s Hospital, Florence, Italy; 2 Department of Medical Basic Sciences, Neurosciences and Sense Organs, University of Bari Aldo Moro, Piazza G. Cesare, 11, 70124 Bari, Italy; 3 IRCCS Fondazione Stella Maris, via dei Giacinti 2, Calambrone, 56128 Pisa, Italy; 4 Metabolic Unit, Meyer Children’s Hospital, Florence, Italy; 5 Present Address: Pediatric Neurology and Centro Clinico Nemo, Fondazione Policlinico Universitario Agostino Gemelli - IRCCS, Rome, Italy

**Keywords:** MTMR5*/SBF1*, Charcot-Marie-Tooth 4B3, Mitochondrial diseases, Next-generation sequencing, Case report

## Abstract

**Background:**

Charcot-Marie-Tooth disease (CMT) type 4B3 (CMT4B3) is a rare form of genetic neuropathy associated with variants in the MTMR5/*SBF1* gene. MTMR5/SBF1 is a pseudophosphatase predicted to regulate endo-lysosomal trafficking in tandem with other MTMRs. Although almost ubiquitously expressed, pathogenic variants primarily impact on the peripheral nervous system, corroborating the involvement of MTMR5/SBF1 and its molecular partners in Schwann cells-mediated myelinization.

**Case presentation:**

We report a case of severe CMT4B3 characterized by early-onset motor and axonal polyneuropathy in an Italian child in absence of any evidence of brain and spine MRI abnormalities or intellectual disability and with a biochemical profile suggestive of mitochondrial disease. Using an integrated approach combining both NGS gene panels and WES analysis, we identified two novel compound heterozygous missense variants in MTMR5/*SBF1* gene, p.R763H (c.2291G > A) and p.G1064E (c.3194G > A). Studies in muscle identified partial defects of oxidative metabolism.

**Conclusion:**

We describe the first case of an early onset severe polyneuropathy with motor and axonal involvement, due to recessive variants in the MTMR5/*SBF1* gene, with no evidence of brain and spine MRI abnormalities, intellectual disability, no clinical and neurophysiological evidences of distal sensory impairment, and rapid neuromuscular deterioration. This report suggests that MTMR5/*SBF1* should be considered in cases of infantile-onset CMT with secondary mitochondrial dysfunction.

**Supplementary Information:**

The online version contains supplementary material available at 10.1186/s12920-021-01001-1.

## Background

Charcot-Marie-Tooth disease (CMT) is one of the most common inherited neurological disorders [1]. It is characterized by chronic motor and/or sensory polyneuropathy with progressive degeneration of muscles in the extremities and by impaired motor and sensory function [2]. Classification has been based on the pattern of inheritance together with the findings of nerve conduction velocity (NCV) studies and nerve biopsy analysis [3], and this has led to the definition of two major subgroups: *axonal* and *demyelinating* forms [4].

CMT4B is among the rarest autosomal recessive demyelinating forms of the disease with fewer than a hundred CMT4B cases reported, mostly coming from countries with high rates of consanguineous marriage [5]. It is subdivided into the three clinically and genetically distinct subtypes CMT4B1, CMT4B2 and CMT4B3, caused by variants in myotubularin-related proteins, namely MTMR2, MTMR13/*SBF2* and MTMR5/*SBF1*, respectively, mainly involved in regulating endolysosomal trafficking [6]. The almost exclusive peripheral nervous system (PNS) involvement seen in CMT4B suggests that myelinating Schwann cells are extremely sensitive to disruption of endolysosomal trafficking [7, 8]. However, it is well established that mutations of myelin components can impact on their axonal counterpart due to rearrangements of protein in the axonal membrane, axonal atrophy and even axonal loss [9].

Mitochondrial diseases (MDs) are heterogeneous conditions resulting from a primary dysfunction of the oxidative phosphorylation (OxPhos) system caused by alterations in either nuclear (nDNA) or mitochondrial (mtDNA) genomes. Peripheral neuropathy is often among the large array of features characterizing MDs associated with defects in genes related to mtDNA maintenance/replication or to ATP synthesis, and in several nDNA-encoded proteins. In most cases, peripheral neuropathy occurs in the setting of multisystemic neurological manifestations, and more rarely as the manifestation at onset [10]. Chronic sensorimotor axonal polyneuropathy is the predominant pattern, whereas demyelinating patterns are less common [11–13]. The fact that MD-like biochemical features are frequent both in non-canonical forms of peripheral neuropathy and in other neuromuscular disorders highlights the critical role played by oxidative metabolism in muscle and nerve function [10, 14].

To date, only five families harboring variants in MTMR5/*SBF1* have been described [15–19] with age at onset of symptoms ranging between a few months and a few years. Clinical and imaging phenotypes are polymorphic: cases may or may not show developmental delay, facial dysmorphisms, and abnormal brain and spinal MRI. Similarly, electrophysiological studies are heterogeneous, revealing mainly demyelinating forms of neuropathy.

We report the case of an Italian girl with progressive and severe infantile-onset axonal motor neuropathy and biochemical features suggestive of mitochondrial disease in whom next-generation sequencing studies allowed us to identify biallelic variants in MTMR5*/SBF1*.

## Case presentation

This 12-year-old Italian girl with an unremarkable family and prenatal history presented bilateral congenital talon-valgus-pronated clubfoot at birth and slight neuromotor developmental delay from the age of 18 months. Her first neurological evaluation at the age of 4 years showed global hypotonia, marked hyperlordosis, anserine gait, Gowers’ sign, easy fatigability, knee valgus and absence of osteotendinous reflexes. Electroneurography disclosed severe motor polyneuropathy with predominant axonal features, diffuse reduction of compound action muscular potentials (CMAP, values under 1.5 mV) and only slight decrement of NCV (38–43 m/s). Sensory action potentials and brain and spinal cord MRI were normal. Gene testing for genomic rearrangements of *PMP22* and punctuate variants in *MNF2* and *GDAP1* were negative. Follow-up electrophysiological evaluations over time showed a slowly progressive reduction of motor action potential amplitude (values under 0.5 mV) with evidence of diffuse denervation activity on electromyography suggesting a neurogenic pattern. Several cognitive evaluations were performed, and all were normal. From the age of 6 years, the patient showed a progressive deterioration, with worsening of ambulation and of respiratory function, to the point of requiring non-invasive positive pressure ventilation, and the development of dysphagia, necessitating G-tube placement. Independent ambulation was lost at the age of 9, when she began needing walking aids; she became wheelchair-dependent at the age of 11. The patient showed no cognitive impairment during the clinical course, and at the latest follow up (age 11), brain and spinal MRI remained normal.

**Fig. 1:**
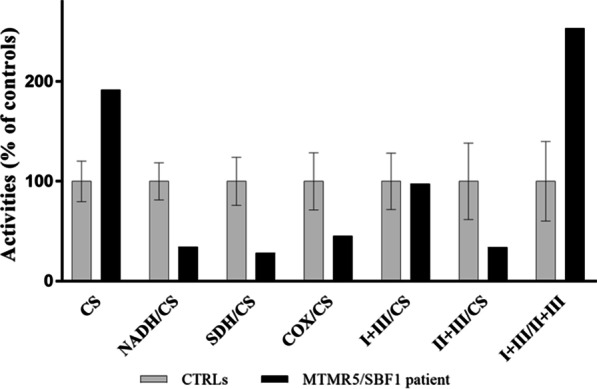
Spectrophotometric analyses of OxPhos complex activities. Respiratory chain enzyme activity measurements showed severe Complex I (NADH), Complex II + III, and Complex IV (COX) defects in skeletal muscle controls. The enzymatic activities of respiratory chain complexes in muscle biopsy from the MTMR5/*SBF1* patient are normalized as ratios to citrate synthase (CS) activity and the data are expressed as percentage of controls (n. 2). All statistical analyses were performed using PRISM® analytical software (GraphPad Inc.)

The progression of the neuromuscular involvement (both clinical and neurophysiological) in this child, in the absence of any metabolic abnormality in blood tests, prompted us to perform a muscle biopsy. This showed a normal histochemical pattern with wiped out areas on oxidative metabolism stains, while NADH staining showed the presence of predominant hypertrophic fibers, suggesting an alteration of the myofibrillar framework. Overall, the results pointed to significant neurogenic muscular atrophy (Additional file 1: Figure S1). Spectrophotometric determination of OxPhos activities in muscle homogenate showed multiple defects of the respiratory chain complexes with high levels of citrate synthetase, an index of mitochondrial proliferation. In particular, these studies [20] showed Complex I (NADH: ubiquinone oxidoreductase), Complexes II + III (succinate: cytochrome *c* reductase) and Complex IV (cytochrome *c* oxidase) activity to be 65, 55 and 66% of normal values, respectively (Fig. 1). Estimation by real-time PCR showed that total mtDNA content was also lower than normal values, as was the mtDNA level in muscle (about 30% of normal control values), whereas the mtDNA/nDNA ratio [21] in cultured skin fibroblasts was normal.

**Fig. 2:**
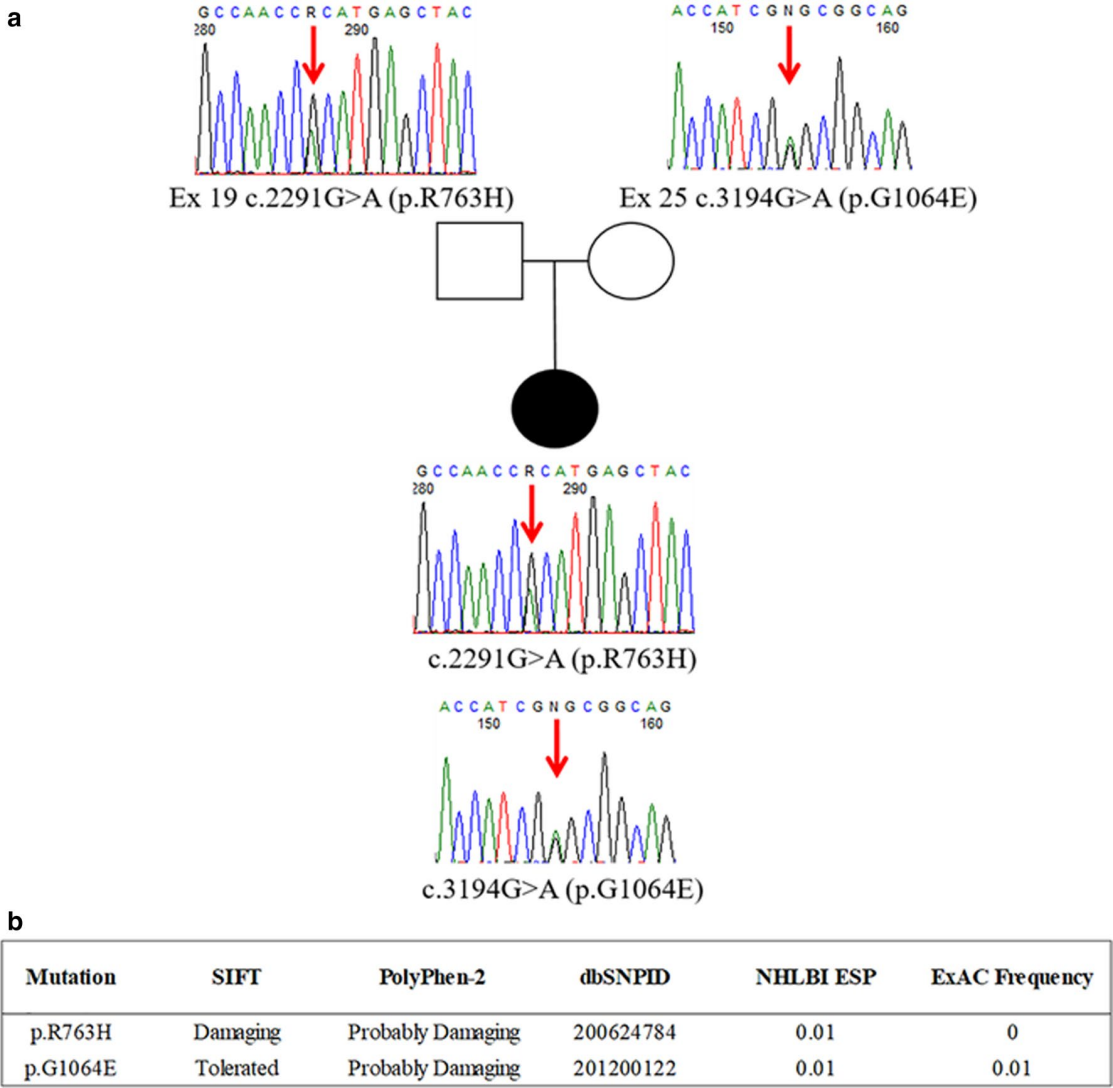
Novel compound heterozygous missense variants in the MTMR5/*SBF1* patient. **a** Familial pedigree and Sanger sequencing data for the MTMR5/*SBF1* patient and her parents demonstrating recessive inheritance of compound heterozygous c.2291G > A (p.R763H) and c.3194G > A (p.G1064E) mutations of MTMR5/*SBF1* (the nomenclature of variant refers to NM_001365819.1). The arrows indicate the variant sites. **b** Predictions of the pathogenic effects of both variants as analyzed using the following prediction tools: SIFT [27]; PolyPhen-2 [28]; dbSNP [29]; NHLBI ESP [30]; GnomAD v3.1 at http://genome.ucsc.edu

Having excluded pathogenic alterations in over 1000 mitochondrial genes by massive gene testing (MitoExome analysis as in [22]), we identified bi-allelic variants in MTMR5/*SBF* (NM_001365819.1) by whole-exome sequencing (WES) of the family trio; this was done by precise filtering and prioritization of over 740 variants in more than 600 rare genes with a customized in-house bioinformatic pipeline using the criteria as reported in [23]. Sanger sequencing confirmed that the patient harbored the p.R763H (c.2291G > A) variant, inherited from her father, and the p.G1064E (c.3194G > A) variant, inherited from her mother (Fig. 2). According to the ACMG guidelines [24], these variants were classified as “uncertain” (evidence of pathogenicity: PM2, PP3) and “likely benign”, respectively, with CADD scores of 29.3 (p.R763H) and 24.2 (p.G1064E). The variants were absent in 175 CMT patients collected in our lab as well as in in-house WES data from 164 cases.

## Discussion and conclusions

**Fig. 3:**
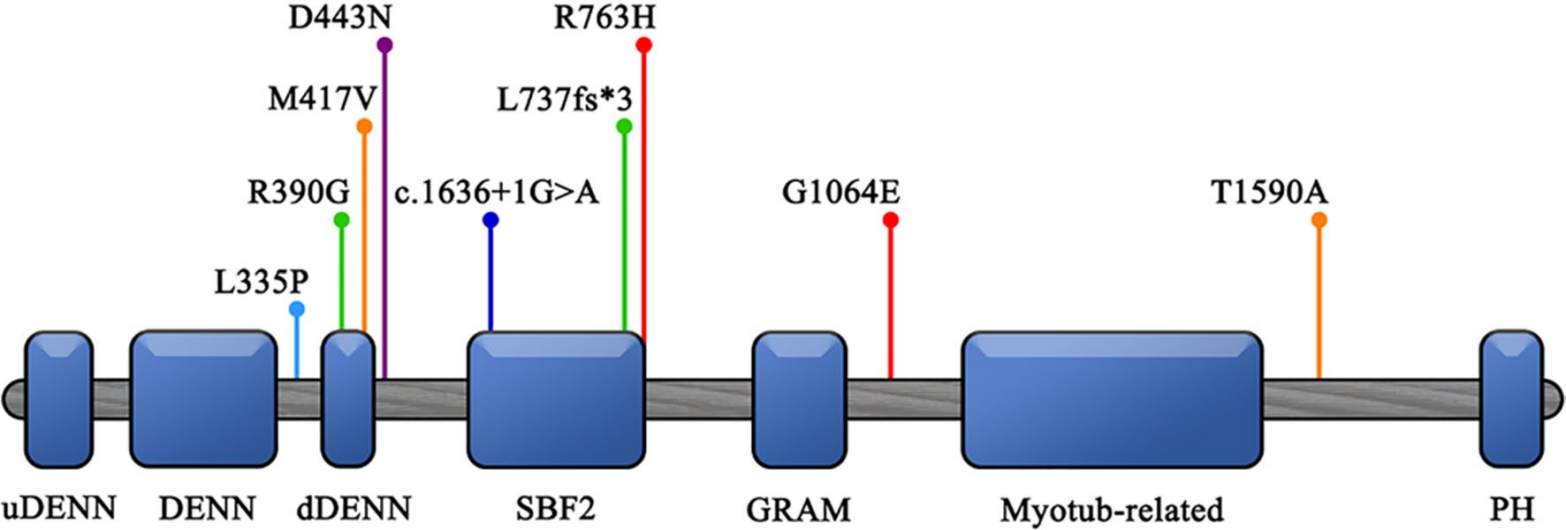
Scheme of the SBF1 protein secondary structure. upstream DENN (uDENN), differentially expressed in normal and neoplastic cells (DENN) and downstream DENN (dDENN) domains, SBF2 domain, Glucosyltransferases, Rab-like GTPase activators and Myotubularins (GRAM) domain, Myotubularin-like phosphatase domain and PH (Pleckstrin Homology) domain. Pins highlight all reported variants: light blue—Romani et al. [19]; green—Manole et al. [17]; orange—Nakhro et al. [18]; purple—Alazami et al. [15]; blue—Flusser et al. [16]; red—our variants

CMT4B3, ranging from pure demyelinating neuropathy with myelin outfolding to axonal forms complicated by multiple cranial involvement, intellectual disability, microcephaly and dysmorphic features, may show various clinical pictures, from mild to more severe. Herein, we further broaden the spectrum of CMT4B3 phenotypes by reporting a case of severe and progressive polyneuropathy with axonal motor involvement in a child without cognitive impairment and with normal MRI findings. We identified two variants in MTMR5*/SBF1* by massive sequencing. The p.R763H mapped on the edge of the SBF1/SBF2 domain of MTMR5 (Fig. 3), a 220-amino acid motif in the middle of both the SBF1 and SBF2 proteins [25]. Although two other variants have been reported in this specific motif [16, 17], its function remains to be ascertained. The second variant, p.G1064E, mapped within a linker region located between the GRAM and myotubularin-like phosphatase domains (Fig. 3).The p.G1064E variant is predicted in silico as tolerated and it might have a moderate impact on the structure/function of the protein. As in our case, less severe missense variants within the linker regions (Fig. 3) were described in three Korean sisters presenting progressive demyelinating sensory-motor neuropathy with myelin outfolding and slow disease progression without cognitive impairment [18]. Conversely, all the remaining CMT4B3 cases harbored pathogenic variants predicting a high functional impact on protein structure [15, 17, 19, 26].

It is interesting that the initial clinical and laboratory investigations in our child suggested a possible mitochondrial role with predominant PNS involvement, a non-canonical, yet at the same time not particularly rare, MD presentation [12]. It is possible that accumulation of toxic metabolites or secondary impairment of the OxPhos machinery and mitochondrial metabolism, or a combination of the two, might affect peripheral nerves and long axons leading to atypical mitochondrial presentations [10–13]. However, these issues require more precise investigations in peripheral nerves.

In conclusion, we identified two novel missense in MTMR5/*SBF1*. This finding underlines the importance of testing this gene in children with clinical evidence of PNS involvement, even if they present no associated neurodevelopmental features or skeletal abnormalities. Whilst our case report further corroborates the importance of WES studies as a first-tier diagnostic method in children with complex neurological phenotypes, it also highlights the presence of secondary features of MD, and thus the need for better studies on the involvement of disturbed oxidative metabolism in chronic inherited neuropathies.

## Supplementary Information

**Additional file 1: Figure S1.** Staining in muscle biopsy from a MTMR5/*SBF1* patient. **a** Muscle-specific staining with hematoxylin and eosin (HE) to show the myofibril morphology; **b** Gomori trichrome staining showing the intermyofibrillar network; **c** ATPase stain at pH 4.3 for type 1 myofibers; **d** NADH staining for respiratory complex I activity; predominant hypertrophic fibers can be seen in the intermyofibrillar network. We used a Zeiss AxioVision microscope and an AxioVision software for data collection. Bar = 50 μ.

## Data Availability

The datasets generated and analyzed during the current study has been registered with the BioProject database with the ID PRJNA733011 (http://www.ncbi.nlm.nih.gov/bioproject/733011). Predictably or probably deleterious scores were determined using an *in-silico *pipeline employing SIFT (https://sift.bii.a-star.edu.sg/), PolyPhen-2 (http://genetics.bwh.harvard.edu/pph2/); dbSNP (https://www.ncbi.nlm.nih.gov/snp/); NHLBI ESP (https://evs.gs.washington.edu/EVS/); GnomAD v3.1 (http://genome.ucsc.edu). Details of the variants analyzed during the current study are deposited in ClinVar (https://www.ncbi.nlm.nih.gov/clinvar/) under the accession codes: RCV001449576.1 (p.Arg763His) and RCV001449656.1 (p.Gly1064Glu). Sanger sequencing was run using the set of primers: Sbf1-19F 5′-TATGGGGATGTGCAGACTCA-3′/Sbf1-19R 5′-ACTCTCCGTGCAGACCTTGT-3′; Sbf1-25F 5′-TCCCTCAGGTATGGATCTGG-3′/Sbf1-25R 5′-CTCCCTGGCCAATGTCAG-3′.

## Abbreviations

CMT: Charcot-Marie-Tooth disease
NCV: Nerve conduction velocity
PNS: Peripheral nervous system
MDs: Mitochondrial diseases
OxPhos: Oxidative phosphorylation
nDNA: Nuclear DNA
mtDNA: Mitochondrial DNA
ACMG: American College of Medical Genetics
