# Supplementary figures and images for: Bi-allelic variants in MTMR5/SBF1 cause Charcot-Marie-Tooth type 4B3 featuring mitochondrial dysfunction
Source: BMC Med Genomics. 2021 Jun 12;14:157. doi: 10.1186/s12920-021-01001-1 (PMC8199524; doi:10.1186/s12920-021-01001-1)

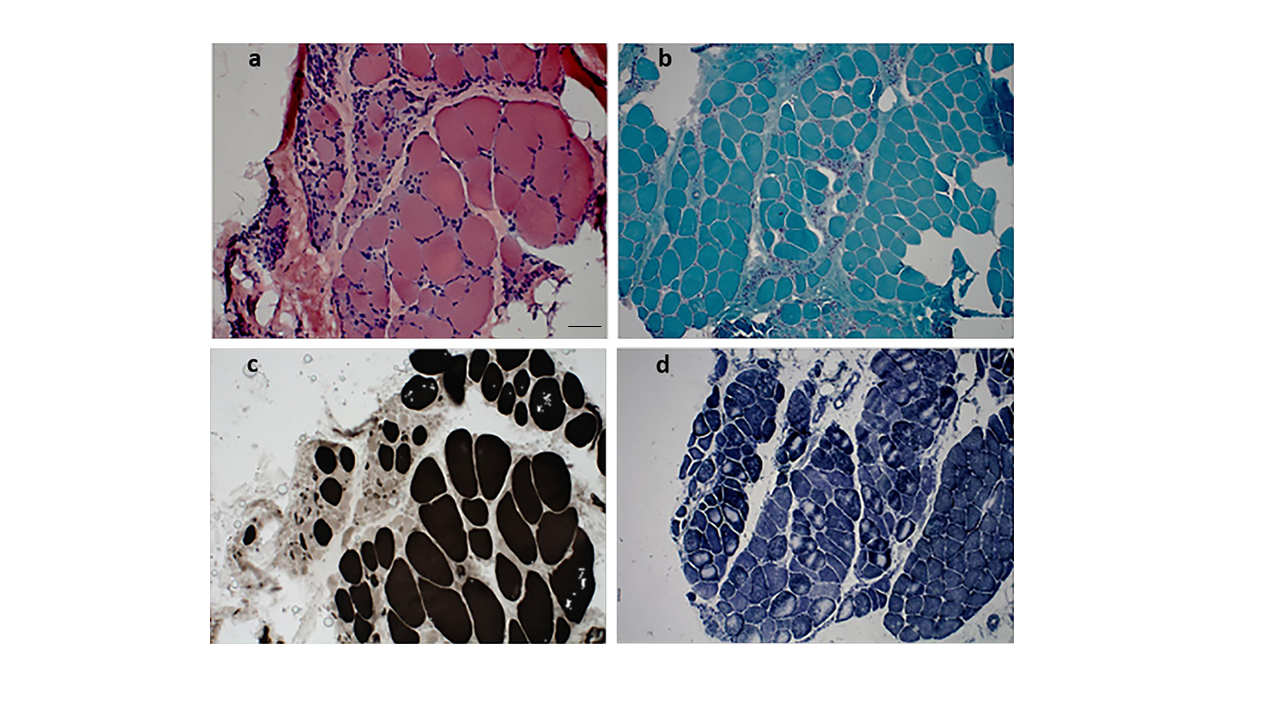

Supplement: Supplementary file 1 — Additional file 1: Figure S1. Staining in muscle biopsy from a MTMR5/SBF1 patient. a Muscle-specific staining with hematoxylin and eosin (HE) to show the myofibril morphology; b Gomori trichrome staining showing the intermyofibrillar network; c ATPase stain at pH 4.3 for type 1 myofibers; d NADH staining for respiratory complex I activity; predominant hypertrophic fibers can be seen in the intermyofibrillar network. We used a Zeiss AxioVision microscope and an AxioVision software for data collection. Bar = 50 μ. [file 12920_2021_1001_MOESM1_ESM.tif]
